# Progress in mechanistic and clinical translational research of endothelin A receptor antagonists in the treatment of diabetic kidney disease: a narrative review

**DOI:** 10.3389/fendo.2026.1808445

**Published:** 2026-05-15

**Authors:** Muqin Li, Chenyang Xie, Yue Qiu, Xiaoyue Li, Guanjun Han, Ning Ma, Guofeng Wang

**Affiliations:** 1 Department of Endocrinology, The First People’s Hospital of Lianyungang, Lianyungang, China; 2 Department of Endocrinology, The Affiliated Lianyungang Hospital of XuZhou Medical University, Lianyungang, China; 3 Department of Endocrinology, Lianyungang Clinical College of Nanjing Medical University., Lianyungang, China; 4 Department of Endocrinology, The First Affiliated Hospital of Kangda College of Nanjing Medical University, Lianyungang, China; 5 Department of Clinical Medicine, Kangda College of Nanjing Medical University, Lianyungang, China; 6 Department of Traditional Chinese Medicine, The First People’s Hospital of Lianyungang, Lianyungang, China

**Keywords:** clinical translation, diabetic kidney disease, endothelin A receptor antagonists, endothelin-1, proteinuria, renal protection

## Abstract

**Background:**

Diabetic kidney disease (DKD) remains a leading cause of end-stage renal disease worldwide. Despite advances in renin–angiotensin–aldosterone system (RAAS) inhibitors, sodium–glucose cotransporter 2 (SGLT2) inhibitors, and mineralocorticoid receptor antagonists (MRAs), substantial residual renal risk persists. Endothelin-1 (ET-1), primarily via endothelin A (ETA) receptor activation, plays a central role in glomerular injury, inflammation, and fibrosis.

**Methodology:**

This narrative review summarizes current evidence on the mechanistic basis and clinical translation of ETA receptor antagonists (ERAs) in DKD. Relevant preclinical and clinical studies were identified through structured literature searches of PubMed, Embase, and Web of Science, focusing on mechanistic insights, therapeutic efficacy, and safety profiles.

**Results:**

Preclinical studies across diabetic models (e.g., streptozotocin-induced and db/db mice) demonstrate that ETA antagonism reduces glomerular hypertension, preserves podocyte integrity, and attenuates inflammatory and fibrotic signaling pathways. In clinical settings, the SONAR trial showed that atrasentan significantly reduced the risk of renal events (hazard ratio ~0.65) and achieved approximately 30–40% reduction in urinary albumin-to-creatinine ratio (UACR) in selected responders. However, ERA therapy is associated with fluid retention and increased heart failure risk. Notably, a biomarker-guided enrichment strategy, based on early albuminuria response during a run-in phase, improved patient selection and benefit–risk balance.

**Conclusion:**

ETA receptor antagonists represent a promising mechanism-based adjunctive therapy for DKD. Their future role is likely to be integrated within combination regimens alongside SGLT2 inhibitors and MRAs, guided by biomarker-driven precision medicine approaches to optimize efficacy while minimizing adverse effects.

## 1 Introduction

Diabetic kidney disease (DKD) is one of the most prevalent microvascular complications of diabetes mellitus and a major contributor to chronic kidney disease (CKD) and end-stage renal disease (ESRD) worldwide. Despite advances in standard-of-care therapies, including renin–angiotensin–aldosterone system (RAAS) inhibitors, sodium–glucose cotransporter 2 (SGLT2) inhibitors, and nonsteroidal mineralocorticoid receptor antagonists (MRAs), a substantial proportion of patients continue to experience progressive renal decline, indicating a significant unmet therapeutic need.

Emerging evidence highlights the endothelin system—particularly endothelin-1 (ET-1) and its interaction with the endothelin A (ETA) receptor—as a key driver of glomerular injury, inflammation, and fibrosis in DKD. Selective ETA receptor antagonists (ERAs) have therefore attracted increasing attention as a targeted therapeutic strategy.

However, the clinical translation of ERAs has been challenging due to safety concerns, particularly fluid retention, as well as variability in patient response. Recent advances in precision medicine, biomarker-guided patient selection, and combination therapy strategies have renewed interest in this drug class.

This review aims to systematically summarize the mechanistic basis, preclinical evidence, and clinical trial data supporting ETA receptor antagonists in DKD, with a focus on translational relevance and future therapeutic integration.

## 2 Methodology

This study is a narrative review aiming to synthesize current evidence on the role of endothelin A receptor antagonists in diabetic kidney disease. A comprehensive literature search was conducted using PubMed, Embase, and Web of Science up to January 2026. Search terms included “diabetic kidney disease,” “endothelin-1,” “ETA receptor antagonist,” “atrasentan,” and “chronic kidney disease.”

Both preclinical and clinical studies were included, focusing on mechanistic insights, pharmacological effects, and clinical outcomes. Priority was given to randomized controlled trials, meta-analyses, and high-impact experimental studies.

The review was conducted in accordance with general principles of narrative synthesis and reporting transparency, aiming to provide an integrated overview of mechanistic and translational evidence.

## 3 Pathogenesis of diabetic kidney disease and the role of the endothelin system

### 3.1 Pathophysiological features of DKD

DKD is characterized by a complex interplay of metabolic, hemodynamic, and inflammatory factors that culminate in progressive renal damage. Hyperglycemia-induced metabolic disturbances are central to DKD pathogenesis, initiating a cascade that includes oxidative stress and chronic inflammation. Elevated glucose levels promote the generation of reactive oxygen species (ROS), which activate pro-inflammatory signaling pathways, leading to the release of cytokines, chemokines, and adhesion molecules that exacerbate renal injury (1, 2). This oxidative stress-mediated inflammation contributes to endothelial dysfunction and disrupts the homeostasis of nitric oxide (NO), a critical regulator of vascular tone and glomerular filtration. Under hyperglycemic conditions, uncoupling of endothelial nitric oxide synthase (eNOS) decreases NO bioavailability and increases peroxynitrite formation, further aggravating oxidative damage (3).

Structurally, DKD manifests as damage to the glomerular filtration barrier, which comprises endothelial cells, the glomerular basement membrane (GBM), and podocytes. Hyperglycemia and associated metabolic derangements induce GBM thickening and mesangial matrix expansion, impairing filtration function (4, 5). Podocyte injury is a hallmark of DKD, involving morphological changes such as hypertrophy, detachment, epithelial-mesenchymal transition, and apoptosis, leading to podocyte loss and proteinuria (6, 7). Recent studies highlight the role of urinary exosomes carrying microRNAs (e.g., miR-145-5p) in mediating podocyte apoptosis via pathways like RhoA/ROCK, revealing novel mechanisms of glomerular injury (8). Additionally, mesangial expansion—characterized by aberrant proliferation of mesangial cells and excessive extracellular matrix deposition—contributes to glomerulosclerosis and progressive renal failure (9).

Interstitial fibrosis and tubular atrophy, secondary to glomerular injury, further compromise renal function. The inflammatory milieu promotes fibrogenic pathways involving transforming growth factor-beta (TGF-β1), connective tissue growth factor (CTGF), and α-smooth muscle actin (α-SMA), leading to extracellular matrix accumulation and scarring (1). Immune cell infiltration, particularly by dendritic cells and T cells, is implicated in both glomerular and tubulointerstitial damage, underscoring the importance of immune-inflammatory crosstalk in DKD progression (10). Moreover, dysregulation of lipid metabolism, including elevated lipoprotein(a) levels, correlates with worsening glomerular pathology and renal function decline, suggesting a contributory role of lipotoxicity (11, 12).

Advances in single-cell RNA sequencing have identified key cell types and molecular pathways involved in DKD, such as the upregulation of mTORC1 activity in proximal tubular cells that drives fibrosis and renal dysfunction—a process ameliorated by sodium-glucose cotransporter 2 inhibitors (SGLT2i) (13). Novel biomarkers, including urinary kidney injury molecule-1 (KIM-1) and plasma miR-193a-3p, show promise in reflecting the extent of renal injury and predicting prognosis (14, 15). Pathological classifications by the Renal Pathology Society and Japanese Renal Pathology Society incorporate glomerular, tubular, and vascular lesions to stratify disease severity and correlate with clinical outcomes (16).

In summary, the pathophysiological features of DKD encompass hyperglycemia-induced metabolic disturbances, oxidative stress, and inflammation that disrupt the glomerular filtration barrier through podocyte injury, mesangial expansion, and basement membrane thickening. These changes are compounded by tubulointerstitial fibrosis and vascular lesions, leading to progressive renal impairment. Understanding these mechanisms provides a foundation for developing targeted therapies and identifying biomarkers for early diagnosis and monitoring of DKD progression.

Importantly, beyond hyperglycemia alone, metabolic dysregulation—particularly insulin resistance and compensatory hyperinsulinemia—acts as an upstream amplifier of endothelin-1 (ET-1) activity, thereby strengthening the mechanistic link between systemic metabolic stress and intrarenal vascular–glomerular injury. In human endothelial systems, insulin can directly stimulate ET-1 production and secretion, supporting the concept that chronic hyperinsulinemia provides a persistent ET-1–driving signal in insulin-resistant states (17). In addition, microvascular studies in humans demonstrate that hyperinsulinemia augments ET-1 protein expression and is associated with impaired vasodilatory responses, consistent with an insulin–ET-1 axis contributing to endothelial dysfunction in cardiometabolic disease (18).

In parallel, obesity-related adipose tissue dysfunction—a hallmark of insulin resistance—creates a chronic low-grade inflammatory milieu characterized by increased free fatty acids and pro-inflammatory adipokines, which further promotes ET-1 pathway activation and vascular dysfunction. JACI Online Contemporary evidence also supports a broader pathophysiological role of ET-1 in obesity and insulin resistance across adipose and skeletal muscle tissues, reinforcing ET-1 as a cardio-kidney-metabolic “nexus” rather than a purely vasoconstrictive mediator (19). This metabolic–endothelin crosstalk can exacerbate intrarenal hemodynamic stress and accelerate glomerular barrier vulnerability (e.g., podocyte stress/injury), thereby promoting persistent proteinuria and progressive renal fibrosis—mechanisms that are not fully addressed by glucose- or blood pressure-centered strategies alone. This framework provides a strong rationale for targeting the endothelin–ETA axis as a mechanism-based therapeutic approach within a broader cardio-kidney-metabolic management paradigm.

### 3.2 ET-1–ETA axis in the pathogenesis of diabetic kidney disease

Endothelin-1 (ET-1) plays a central and multifaceted role in the pathogenesis of diabetic kidney disease (DKD), acting predominantly through activation of the endothelin A receptor (ETAR) to promote glomerular injury, inflammation, and fibrosis.

Under diabetic conditions, ET-1 synthesis is significantly upregulated in renal endothelial cells, podocytes, and mesangial cells. The biosynthesis of ET-1 involves transcription of preproendothelin-1, which is cleaved to big endothelin-1 and subsequently converted into active ET-1 by endothelin-converting enzyme (ECE) (20, 21). This process is markedly enhanced by hyperglycemia, oxidative stress, and inflammatory stimuli, leading to sustained intrarenal ET-1 accumulation.

ET-1 exerts its biological effects through two G protein–coupled receptors, ETAR and ETBR, which exhibit distinct functional roles and tissue distributions (21). While ETBR contributes to vasodilation and ET-1 clearance, ETAR activation predominantly mediates vasoconstriction, cellular proliferation, inflammation, and fibrosis, making it the principal driver of pathological processes in DKD.

A key pathogenic mechanism involves ETAR-mediated glomerular injury. Activation of ETAR on mesangial cells promotes proliferation and excessive extracellular matrix deposition, leading to mesangial expansion and glomerulosclerosis (22). Concurrently, ET-1 signaling in podocytes disrupts cytoskeletal integrity, induces apoptosis, and impairs slit diaphragm function, resulting in increased glomerular permeability and proteinuria (23).

Beyond structural damage, ET-1 is a potent regulator of inflammatory and fibrotic signaling pathways. ETAR activation stimulates the production of pro-inflammatory cytokines, including interleukin-6 (IL-6) and tumor necrosis factor-α (TNF-α), and upregulates profibrotic mediators such as TGF-β1 and platelet-derived growth factor-BB (PDGF-BB), thereby driving tubulointerstitial fibrosis and chronic inflammation (24–26). In addition, ET-1 enhances immune-mediated injury, including the activation of tissue-resident immune cells that further exacerbate glomerulosclerosis (27).

ET-1 also contributes to intrarenal hemodynamic dysregulation. Through ETAR-mediated vasoconstriction, ET-1 increases glomerular capillary pressure and exacerbates hyperfiltration, accelerating mechanical stress on the glomerular filtration barrier (28). Importantly, ET-1 signaling interacts with other key pathogenic pathways, including the renin–angiotensin–aldosterone system (RAAS) and oxidative stress cascades, amplifying renal injury in DKD (29, 30).

Emerging evidence further highlights the role of ET-1 in metabolic–vascular crosstalk. Insulin resistance and hyperinsulinemia can directly enhance ET-1 production, while adipose tissue inflammation promotes endothelin pathway activation, positioning ET-1 as a critical mediator linking metabolic dysfunction to renal injury within the cardio–kidney–metabolic axis (17–19).

Finally, recent modeling studies of endothelin kinetics suggest that selective ETAR antagonism reduces pathological ET-1 signaling while variably influencing ETBR activity depending on drug selectivity and concentration, providing important insights into therapeutic optimization (31).

Collectively, the ET-1–ETA signaling axis integrates hemodynamic, inflammatory, fibrotic, and metabolic mechanisms that drive DKD progression. This mechanistic centrality provides a strong rationale for targeting ETAR as a therapeutic strategy to achieve incremental renal protection beyond current standard-of-care therapies.

## 4 Pharmacological mechanisms and targets of endothelin A receptor antagonists

**Figure 1:**
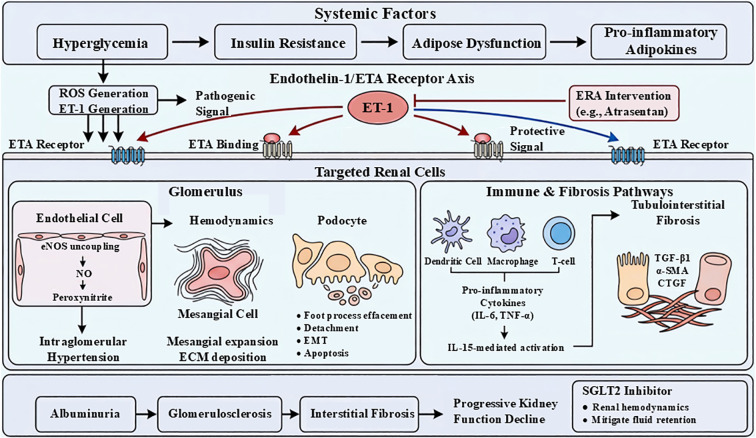
The comprehensive pathogenesis of diabetic kidney disease (DKD) and the mechanistic targets of endothelin A (ETA) receptor antagonists. Metabolic disturbances, including hyperglycemia, insulin resistance, and adipose dysfunction, increase oxidative stress (ROS) and upregulate endothelin-1 (ET-1) production. ET-1 signaling via ETA receptors induces endothelial dysfunction (reduced nitric oxide bioavailability), mesangial expansion with extracellular matrix accumulation, and podocyte injury (foot process effacement, detachment, epithelial–mesenchymal transition, and apoptosis). These changes are accompanied by immune activation and release of pro-inflammatory cytokines (IL-6, TNF-α) and profibrotic mediators (TGF-β1, α-SMA, CTGF), promoting tubulointerstitial fibrosis. Collectively, these processes lead to albuminuria, glomerulosclerosis, and progressive renal dysfunction. Selective ETA receptor antagonists (e.g., atrasentan) mitigate these pathological pathways, while combination therapy with SGLT2 inhibitors provides additional renoprotective effects.

The mechanisms of endothelin A receptor antagonism and their therapeutic implications in DKD are illustrated in **Figure 1**.

### 4.1 Preclinical evidence supporting ETA antagonism

The mechanistic rationale for targeting the endothelin system in DKD has been robustly supported by a wealth of recent preclinical models. Experimental studies utilizing both highly selective ETA receptor antagonists and dual-acting antagonists have demonstrated significant improvements in glomerular hemodynamics, structural integrity, and inflammatory profiles (32).

Highly selective ETA receptor antagonists, such as atrasentan, have shown profound renoprotective effects across different types of diabetes models (33, 34). In a spontaneous Type 2 diabetes model using *db/db* mice (driven by a *Lepr^db* mutation), Ander Vergara et al. (2022) Addition of the ERA atrasentan to treatment with an SGLT2 inhibitor and RAS blockade also enhanced kidney protection in a mouse model of type 2 diabetes, as evidenced by a greater reduction in podocyte loss than that achieved with an ERA, SGLT2 inhibitor, or RAS blockade monotherapy or duo therapy with any combination of these three drug classes, suggesting that these three drug classes have partly non-overlapping mechanisms of action and may be beneficial in combination. Mechanistically, specific ETA blockade downregulated the local renal renin-angiotensin system (RAS), particularly decreasing angiotensin II levels and AT1R expression, thereby suppressing downstream inflammatory and fibrotic pathways (33).

Furthermore, the protective effects of ETA receptor antagonists extend to Type 1 diabetes. In a STZ-induced Type 1 diabetic WKY rat model, Alessandro Cosenzi et al. (2003) revealed that bosentan (Non-selective ETA/ETB antagonist) significantly prevented the increase in urinary protein excretion and that of renal immunoreactive collagen I, fibronectin, and TGF-beta induced by diabetes without reducing blood pressure. This renoprotection was closely associated with the modulation of renal metabolic reprogramming, improvement of mitochondrial function, and the direct inhibition of the profibrotic TGF-β/Smad3 signaling pathway (35).

Recognizing the complex pathophysiology of DKD, another preclinical studies have also explored the efficacy of dual receptor antagonists. Macitentan, a dual ETA/ETB receptor antagonist, was evaluated by S Chakrabarti et al. (2012) in the db/db mice. The study found that diabetic animals showed hyperglycemia, increased urinary albumin, augmented serum creatinine levels along with increased FN and collagen protein and NF-κB activation. Treatment with macitentan attenuated such abnormalities, which means that plays a significant role in the pathogenesis of chronic complications in type 2 diabetes (36).

Similarly, Darusentan (selective ETA antagonist) was investigated in the streptozotocin-induced diabetic rats by B Hocher et al. (2001). The study demonstrated that treatment with darusentan reduced the daily protein excretion by >50% 36 weeks after diabetes induction, compared with non-treated rats (32). Klaus Witte also found that Darusentan reduced 24-h blood pressure but did not restore the endothelium-dependent vasorelaxation. These studies utilizing both highly selective ETA receptor antagonists and dual-acting antagonists plays a significant role in the pathogenesis of chronic complications in type 2 diabetes (37).

To systematically summarize the translational value of these recent findings and address the heterogeneity of DKD models, **Table 1** provides a comprehensive overview of these key preclinical studies, detailing the animal models, methods of disease induction, specific interventions, and primary molecular mechanisms.

**Table 1:** Summary of preclinical studies evaluating ETA receptor antagonists in DKD.

Intervention (drug)	Animal model	Species	Method of disease induction	Key findings and mechanisms
Bosentan (Dual ETA/ETB antagonist)	STZ-induced Type 1 diabetic kidney disease	WKY Rats	Single high-dose intraperitoneal injection of streptozotocin (STZ)	Key Findings: Bosentan has prevented the increase in urinary protein excretion and that of renal immunoreactive collagen I, fibronectin, and TGF-beta induced by diabetes without reducing blood pressure.
Atrasentan (Selective ETA antagonist)	Type 2 diabetic kidney disease	db/db mice	Spontaneous genetic model (leptin receptor deficiency)	Key Findings: Addition of the ERA atrasentan to treatment with an SGLT2 inhibitor and RAS blockade also enhanced kidney protection, as evidenced by a greater reduction in podocyte loss than that achieved with an ERA, SGLT2 inhibitor,or RAS blockade monotherapy or duo therapy with any combination of these three drug classes.
Macitentan (Dual ETA/ETB antagonist)	Type 2 diabetic kidney disease	db/db mice	Spontaneous genetic model (leptin receptor deficiency)	Key Findings: Treatment with macitentan attenuated hyperglycemia induced hyperglycemia, increased urinary albumin, augmented serum creatinine levels along with increased FN and collagen protein and NF-κB activation.
Darusentan (Selective ETA antagonist)	STZ-induced Type 2 diabetic kidney disease	Rats	High-fat diet combined with low-dose STZ inductio	Key Findings: Treatment with darusentan reduced the daily protein excretion by >50% 36 weeks after diabetes induction, compared with non-treated rats.
Darusentan (Selective ETA antagonist)	Non-obese spontaneous type 2 diabetes model	GK Rats (Goto–Kakizaki Rat)	Selective inbreeding of Wistar rats with impaired glucose tolerance	Key Findings: Darusentan led to a reduction in 24-h blood pressure but did not restore the endothelium-dependent vasorelaxation, suggest that an activated endothelin pathway may contribute to elevated BP but is not involved in vascular dysfunction in this animal.

### 4.2 Pharmacological properties and classification of endothelin receptor antagonists

ERAs represent a class of therapeutic agents targeting the endothelin system, which plays a pivotal role in regulating vascular tone and contributes to the pathophysiology of conditions such as pulmonary arterial hypertension (PAH), resistant hypertension, and diabetic kidney disease. ERAs are classified based on their receptor selectivity and distinct pharmacokinetic/pharmacodynamic profiles into two main categories: selective ETA antagonists and non-selective antagonists that block both ETA and ETB receptors.

Selective ETAR antagonists, including atrasentan and ambrisentan, preferentially inhibit the ETAR, which mediates vasoconstriction and proliferative effects induced by endothelin-1 (ET-1). This selectivity aims to preserve ETB receptor-mediated vasodilation and ET-1 clearance, potentially reducing adverse effects associated with ETB blockade. For instance, clazosentan—a potent selective ETA antagonist—has demonstrated efficacy in preventing cerebral vasospasm following aneurysmal subarachnoid hemorrhage. Its pharmacokinetic profile is characterized by intermediate clearance, dose-proportional exposure, and elimination independent of drug-metabolizing enzymes, relying primarily on hepatic uptake transporters (OATP1B1/1B3). Similarly, ambrisentan exhibits high affinity for ETARs and has been widely used in PAH treatment, showing favorable efficacy and safety in clinical trials (38).

Non-selective ERAs, such as bosentan, inhibit both ETA and ETB receptors. While bosentan has been extensively studied and approved for PAH treatment, demonstrating improvements in exercise capacity and hemodynamics, its blockade of ETB receptors may impair ET-1 clearance and vasodilatory pathways, potentially contributing to side effects like fluid retention and hepatotoxicity (38).

The pharmacokinetics and pharmacodynamics of ERAs vary significantly among agents. Aprocitentan, a recently approved dual ETA/ETB antagonist for resistant hypertension, achieves maximum plasma concentration within 4–5 hours post-administration and has an effective half-life of approximately 41 hours. It undergoes hepatic and renal metabolism primarily via UGT1A1- and UGT2B7-mediated pathways and is highly protein-bound, mainly to albumin (39). Its pharmacodynamic action involves inhibition of ET-1-mediated vasoconstriction, contributing to sustained blood pressure reduction (40). Notably, aprocitentan also exhibits carbonic anhydrase inhibitory activity, which may enhance its antihypertensive efficacy by modulating renal and vascular physiology (41).

Other ERAs, like macitentan, have been associated with elevated hepatic enzymes in some patients. Pharmacogenetic factors, such as polymorphisms in CYP2C8 and CYP2C9 enzymes, influence drug metabolism and hepatotoxicity risk, highlighting the importance of individualized pharmacological counseling and monitoring during ERA therapy (42).

Beyond receptor affinity and metabolic pathways, pharmacological profiles of ERAs also include differences in bioavailability, tissue distribution, and safety. Selective ETA antagonists generally exhibit fewer hepatic side effects and less fluid retention compared to non-selective agents, which is crucial for long-term safety in chronic conditions like diabetic kidney disease and resistant hypertension (38–41).

### 4.3 Protective effects of endothelin receptor antagonists on glomerular structure and function

ERAs, particularly selective ETAR blockers like atrasentan, have demonstrated significant protective effects on glomerular structure and function in DKD and other proteinuric CKD. A key mechanism involves the inhibition of mesangial cell proliferation and collagen deposition, which are critical contributors to glomerulosclerosis and progressive renal fibrosis. Excessive mesangial expansion and extracellular matrix accumulation disrupt normal glomerular architecture, impairing filtration capacity. ERAs mitigate these pathological processes by antagonizing endothelin-1 (ET-1)-mediated signaling pathways that promote mesangial cell activation and fibrogenesis, thereby preserving glomerular structural integrity (25).

In addition to protecting mesangial cells, ERAs play a crucial role in safeguarding podocytes—specialized epithelial cells that form the slit diaphragm and maintain the glomerular filtration barrier. Podocyte injury and loss are hallmark features of DKD and other glomerular diseases, resulting in increased permeability and proteinuria. By blocking ETARs, ERAs reduce podocyte stress and apoptosis, maintaining the integrity of the filtration barrier. This preservation of podocyte function is essential for preventing the onset and progression of proteinuria, a key clinical marker of glomerular damage and predictor of renal outcomes (43).

Furthermore, ERAs contribute to reducing proteinuria and glomerular hyperfiltration, two interrelated phenomena that exacerbate kidney injury. Proteinuria reflects increased permeability of the glomerular barrier and serves as both a marker and mediator of progressive renal damage. Glomerular hyperfiltration, often observed in early DKD, increases intraglomerular pressure and shear stress, accelerating injury to glomerular cells. ERA treatment reduces glomerular perfusion pressure, alleviating hyperfiltration and its deleterious effects. The SONAR trial, a landmark phase 3 study, demonstrated that long-term treatment with atrasentan significantly decreased proteinuria and slowed CKD progression in patients with DKD, highlighting the clinical relevance of these protective mechanisms (43, 44).

### 4.4 Mechanisms of ERA in anti-inflammation and anti-fibrosis

Endothelin receptor antagonists (ERAs), particularly those targeting the ETAR, have emerged as promising agents for modulating inflammatory and fibrotic processes implicated in DKD and CKD. A key anti-inflammatory mechanism of ERAs involves the suppression of pro-inflammatory cytokine expression. Endothelin-1 (ET-1), acting primarily through ETAR, induces the production of several pro-inflammatory mediators, including TNF-α, interleukins (e.g., IL-6, IL-12p40, IL-17), and chemokines that contribute to renal inflammation. In models of inflammatory diseases, selective ETAR antagonists like ambrisentan significantly reduce the expression of these cytokines and attenuate immune cell activation—including dendritic cells and T cells—thereby dampening the inflammatory milieu (45). Moreover, ETAR blockade decreases the infiltration of inflammatory cells such as macrophages and T lymphocytes into the kidney, which are key players in the progression of renal injury (46). This immunomodulatory effect is partly mediated through inhibition of intracellular signaling pathways, including the extracellular signal-regulated kinase (ERK) pathway and transcription factors like GATA3, which regulate cytokine production and immune cell function.

In addition to their anti-inflammatory effects, ERAs exert potent anti-fibrotic actions by blocking fibrogenic signaling cascades that lead to renal interstitial fibrosis, a hallmark of progressive DKD. ET-1 promotes the activation and proliferation of fibroblasts and myofibroblasts, stimulates extracellular matrix (ECM) production, and induces epithelial-to-mesenchymal transition (EMT), all contributing to tubulointerstitial fibrosis. ERAs inhibit these processes by antagonizing ETAR-mediated signaling, thereby reducing collagen deposition and fibrotic remodeling in the kidney (47, 48). Experimental studies have shown that ETAR antagonism attenuates the expression of profibrotic markers and decreases perivascular collagen accumulation, correlating with improved renal function and histopathology (49). Notably, selective ETAR antagonists such as atrasentan and sparsentan (a dual angiotensin II type 1 receptor and ETAR blocker) demonstrate superior efficacy in reducing proteinuria and fibrosis compared to angiotensin receptor blockers alone, highlighting the critical role of endothelin signaling in fibrogenesis (49, 50). Furthermore, the anti-fibrotic benefits of ERAs may be enhanced when combined with sodium-glucose cotransporter 2 inhibitors (SGLT2i), which mitigate ERA-associated side effects like fluid retention and potentiate renal protection (25, 51).

### 4.5 Metabolic modulation by ETA receptor antagonism

Beyond their well-established anti-inflammatory and anti-fibrotic effects within the kidney, ETA receptor antagonists exert important metabolic actions that are highly relevant to the pathophysiology of type 2 diabetes–associated DKD. Increasing evidence indicates that endothelin-1 (ET-1) signaling contributes directly to systemic insulin resistance by impairing insulin-mediated glucose uptake in skeletal muscle and disrupting endothelial insulin signaling, thereby limiting insulin delivery to peripheral tissues (52, 53). Selective ETAR antagonism has been shown to improve insulin sensitivity in both experimental models and clinical studies, as reflected by reductions in fasting insulin levels and homeostasis model assessment of insulin resistance (HOMA-IR), suggesting that blockade of ETA signaling partially restores metabolic–vascular coupling (54, 55).

Adipose tissue inflammation represents another critical metabolic target of ETAR antagonism. In insulin-resistant and obese states, ET-1 expression is upregulated within adipose tissue, where it promotes macrophage infiltration, pro-inflammatory cytokine release, and adipocyte dysfunction. Experimental studies demonstrate that ETAR blockade attenuates adipose tissue inflammation by reducing TNF-α, IL-6, and other inflammatory mediators, thereby improving adipokine profiles and systemic insulin responsiveness. This anti-inflammatory effect in adipose tissue complements the renoprotective actions of ETA antagonists by mitigating upstream metabolic stressors that drive glomerular and vascular injury.

**Figure 2:**
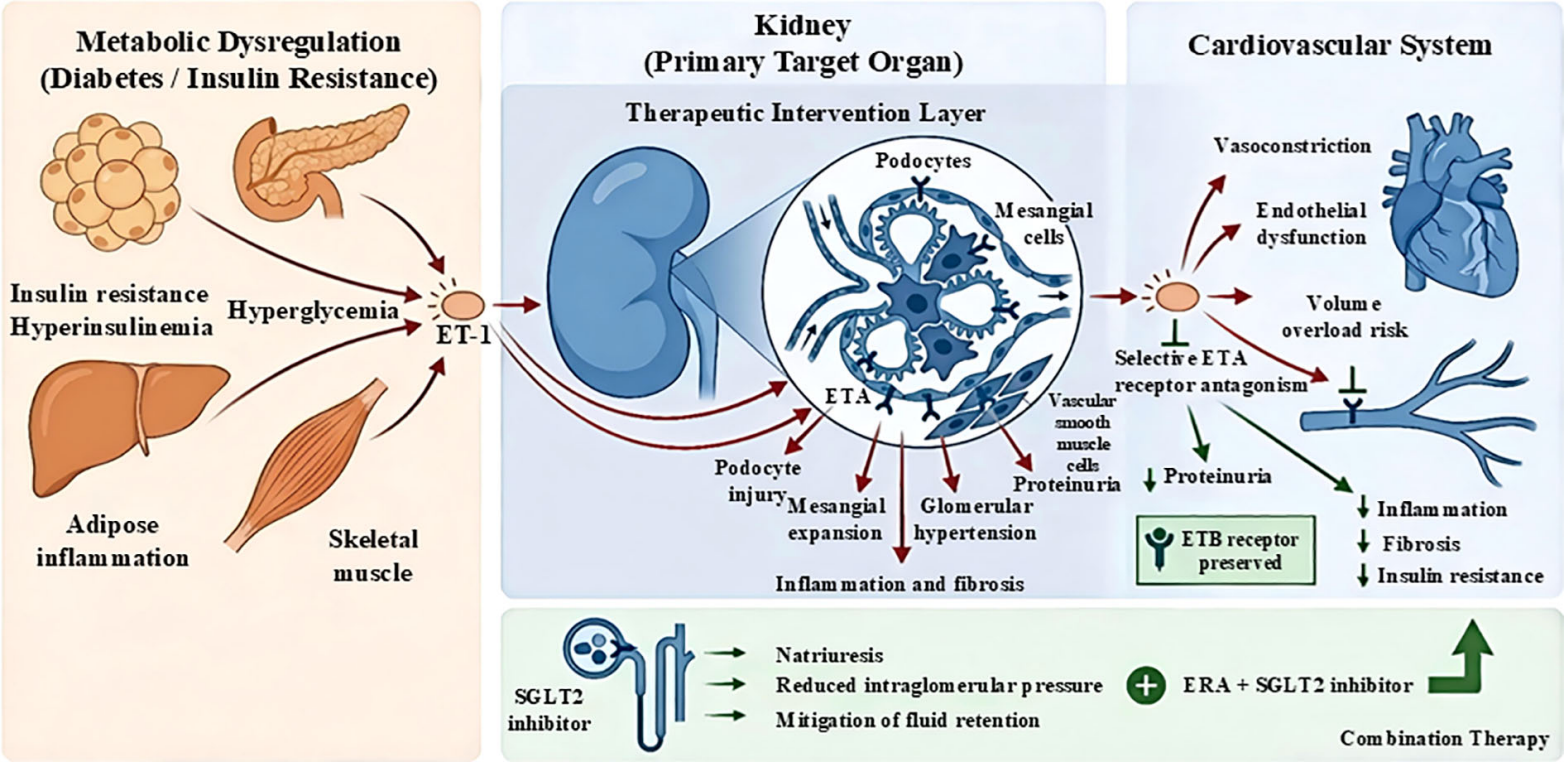
The endothelin-1–ETA axis as a central cardio–kidney–metabolic link in diabetic kidney disease. Metabolic dysregulation associated with diabetes and insulin resistance promotes ET-1 overproduction through hyperglycemia, hyperinsulinemia, adipose tissue inflammation, and skeletal muscle dysfunction. The kidney serves as the primary target organ, where ET-1–mediated ETA activation induces podocyte injury, mesangial expansion, glomerular hypertension, inflammation, and fibrosis. Selective ETA receptor antagonism attenuates renal injury while preserving ETB receptor–mediated protective effects. Combination therapy with sodium–glucose cotransporter 2 (SGLT2) inhibitors further reduces intraglomerular pressure, enhances natriuresis, and mitigates fluid retention, thereby integrating renal, cardiovascular, and metabolic protection.

These metabolic effects provide a compelling explanation for the preferential efficacy of ETAR antagonists observed in patients with type 2 diabetes mellitus (T2DM) compared with type 1 diabetes. T2DM is characterized by insulin resistance, hyperinsulinemia, and adipose tissue dysfunction—conditions that are closely linked to ET-1 overactivation. By contrast, in type 1 diabetes, where absolute insulin deficiency predominates, the metabolic contribution of the ET-1–ETA axis may be less pronounced (22). Consequently, ETAR antagonism may confer dual benefits in T2DM by simultaneously attenuating renal injury and improving systemic metabolic homeostasis, positioning this drug class as a mechanistically targeted therapy within the broader cardio–kidney–metabolic framework of DKD management (**Figure 2**).

## 5 Clinical research progress of endothelin A receptor antagonists

### 5.1 Overview of key clinical trials

The SONAR (Study of Diabetic Nephropathy with Atrasentan) trial represents a pivotal clinical investigation evaluating the efficacy of the selective endothelin A receptor antagonist (ERA) atrasentan in patients with type 2 diabetes and DKD. This randomized, double-blind, placebo-controlled trial enrolled participants with moderate CKD, defined by an estimated glomerular filtration rate (eGFR) between 25 and 75 mL/min/1.73 m² and significant albuminuria (UACR 300–5000 mg/g). The primary composite endpoint—doubling of serum creatinine, progression to kidney failure (dialysis, transplantation), or death from kidney failure—was significantly reduced with atrasentan treatment, demonstrating its renoprotective effects when added to RAAS blockade therapy (22, 56)(**Table 2**).

**Table 2:** Summary of key clinical trials evaluating ERAs in diabetic and chronic kidney disease.

Trial name and registry number	Phase and status	Study population	Intervention	Quantitative outcomes and safety profile
SONAR (NCT00120328)	Phase 3(Completed)	Type 2 diabetes with CKD (eGFR 25-75 mL/min/1.73m²; UACR 300-5000 mg/g) on max RAASi.	Atrasentan 0.75 mg/day vs. Placebo (after an enrichment phase selecting for ≥30% UACR reduction).	Efficacy: Primary composite renal endpoint reduced by 35% (HR 0.65; p=0.0047).Safety: Heart failure hospitalization rate was 3.5% (Atrasentan) vs 2.6% (Placebo).
ASCEND (NCT00120328)	Phase 3(Terminated early)	Type 2 diabetes with CKD with eGFR ≥15 mL/min/1.73m² and UACR > 309 mg/g.	Atrasentan 25 and 50 mg/day vs. Placebo.	Efficacy: In patients treated with avosentan 25 mg/d, 50 mg/d, and placebo, the median reduction in albumin-to-creatinine ratio was 44.3, 49.3, and 9.7%. Adverse events led to discontinuation of trial medication significantly more often for avosentan than for placebo (19.6 and 18.2 versus 11.5% for placebo).
ZENITH-CKD(NCT06087835)	Phase 2b(Completed)	CKD of different aetiologies; eGFR 20–90 mL/min;UACR >700 mg/g or UPCR >1000 mg/g.	Zibotentan (0.25 mg or 0.75 mg) + Dapagliflozin (10 mg) vs. Dapagliflozin alone.	Efficacy: UACR reduced by an additional 33.7% (high-dose) and 27.0% (low-dose) compared to Dapa alone.Safety: Fluid retention events were 18.4% (high-dose), 8.8% (low-dose), and 7.9% (Dapa alone).

Atrasentan also markedly reduced albuminuria, a key surrogate marker of renal damage, and improved blood pressure and lipid profiles. These effects collectively contributed to slowing renal dysfunction in DKD. However, sex-specific differences were noted: although female participants exhibited greater renal protection, they also experienced a higher incidence of heart failure events compared to males, underscoring the need for tailored dosing strategies to optimize the benefit–risk balance (57). Fluid retention remains a concern with ERA therapy; nevertheless, atrasentan demonstrated a more manageable safety profile compared to earlier agents like avosentan and bosentan, with fewer fluid overload-related adverse events (58, 59).

Mechanistically, atrasentan’s benefits are attributed to its selective blockade of the ETAR, which mediates vasoconstriction, inflammation, and fibrosis in the kidney. By inhibiting ETA signaling, atrasentan reduces glomerular hypertension, podocyte injury, and mesangial proliferation, thereby preserving glomerular filtration barrier integrity and reducing proteinuria. Preclinical models support these findings, showing that atrasentan restores podocyte number and decreases mesangial matrix expansion (23). Additionally, atrasentan has been shown to reduce insulin resistance, further supporting its multifaceted role in DKD management (60).

Despite these promising outcomes, the SONAR trial highlights the necessity for careful patient selection and monitoring, particularly for fluid retention and heart failure risk. SONAR establishes atrasentan as a potent adjunct therapy for DKD, capable of reducing proteinuria and delaying progression to end-stage kidney disease (ESKD) when used judiciously (61). Ongoing efforts aim to optimize dosing regimens and explore combination therapies to mitigate adverse effects while maximizing renal protection.

Building on the success of selective ETAR antagonists, recent research has explored combination therapies to enhance efficacy and safety in CKD, including DKD. The ZENITH-CKD trial investigates the potential synergistic effects of combining an ETAR antagonist with sodium–glucose cotransporter 2 (SGLT2) inhibitors—a class of agents with established renal and cardiovascular benefits in DKD and non-diabetic CKD (25, 62).

SGLT2 inhibitors lower intraglomerular pressure and reduce albuminuria while conferring cardiovascular protection. Importantly, their diuretic and natriuretic effects may counterbalance fluid retention associated with ERA therapy, potentially improving the tolerability and safety profile of ETAR blockade (25, 62). Preclinical and early-phase clinical studies have shown that co-administration of a low-dose ETA-selective ERA (e.g., zibotentan) with an SGLT2 inhibitor (e.g., dapagliflozin) enhances albuminuria reduction and mitigates fluid retention in CKD patients, suggesting complementary mechanisms (28).

The rationale for this combination stems from the distinct yet overlapping pathophysiological pathways targeted by each agent. While ERAs primarily inhibit endothelin-1–mediated vasoconstriction, inflammation, and fibrosis, SGLT2 inhibitors improve metabolic parameters, reduce tubular workload, and attenuate renal hypoxia. This multi-targeted approach addresses the complex mechanisms driving DKD progression more effectively than monotherapy (62).

Furthermore, this strategy aligns with emerging standards advocating multi-drug regimens to achieve additive or synergistic benefits in CKD management. Recent reviews emphasize that such approaches can reduce the risk of kidney failure and cardiovascular events beyond what is achievable with single agents, while careful monitoring and individualized treatment can minimize adverse effects (62).

Ongoing Phase 3 trials, including ZENITH-CKD, are rigorously evaluating the efficacy, safety, and optimal dosing of ETAR antagonists in combination with SGLT2 inhibitors. These studies aim to determine whether therapeutic synergy translates into improved clinical outcomes, such as sustained reduction in proteinuria, slower eGFR decline, and decreased incidence of ESKD. The results will significantly influence the future clinical application of ERAs, potentially expanding their use in diabetic and non-diabetic CKD populations (62).

### 5.2 Differential efficacy of ERAs in various DKD patient populations

#### 5.2.1 Differential responses in type 1 and type 2 diabetes patients

ERAs, particularly selective ETAR antagonists like atrasentan, have shown promise in treating DKD; however, their efficacy and safety profiles vary between type 1 (T1DM) and type 2 diabetes mellitus (T2DM) populations. Most clinical trials and meta-analyses have focused predominantly on T2DM patients with CKD, reflecting the higher global prevalence of T2DM and its associated nephropathy. For example, the SONAR trial and subsequent meta-analyses demonstrated that atrasentan effectively reduces albuminuria and slows renal function decline in T2DM patients with DKD, with an optimal dose of 0.75 mg/day balancing renoprotection and fluid retention risk (22, 63). Albuminuria reduction, a key surrogate marker for renal protection, is well-established with atrasentan in T2DM, alongside benefits in blood pressure and lipid profiles (43).

In contrast, data on ERA efficacy in T1DM patients remain limited. The pathophysiology of DKD differs between T1DM and T2DM: T1DM involves autoimmune-mediated beta-cell destruction leading to insulin deficiency, whereas T2DM is characterized by insulin resistance and metabolic syndrome components. These differences may influence endothelin-1 (ET-1) system activation and response to ERAs. Although experimental models suggest that ET-1 contributes to podocyte injury and glomerulosclerosis in both types, clinical evidence supporting ERA benefits in T1DM is sparse (64). Moreover, insulin resistance—more prevalent in T2DM—is associated with worse cardiorenal outcomes and may be ameliorated by ERAs, as evidenced by reductions in HOMA-IR with atrasentan treatment (60).

Safety profiles also differ: T2DM patients, especially those with advanced CKD, are at higher risk of fluid retention and congestive heart failure with ERA therapy, necessitating vigilant monitoring and dose optimization (63). Although T1DM patients may have a lower baseline risk for these adverse events, this requires confirmation in dedicated studies. Thus, while ERAs are effective in reducing albuminuria and slowing DKD progression in T2DM, their role in T1DM remains to be fully elucidated, highlighting the need for clinical trials specifically targeting T1DM populations to assess efficacy, safety, and optimal dosing.

#### 5.2.2 Applicability and efficacy across different stages of renal function

The stage of kidney impairment significantly influences the therapeutic applicability and efficacy of ERAs in DKD. Most clinical trials have enrolled patients with moderate CKD (eGFR 25–75 mL/min/1.73 m²) and significant albuminuria, indicative of active renal injury (63, 64). In these populations, ERAs like atrasentan have demonstrated substantial reductions in albuminuria and delayed progression to composite renal endpoints, including doubling of serum creatinine and end-stage kidney disease (ESKD) (63). The SONAR trial, for instance, showed that atrasentan reduced the risk of kidney events with a tolerable safety profile in T2DM patients with CKD stages 3–4 (43).

However, efficacy and safety data in patients with advanced CKD (eGFR < 25 mL/min/1.73 m²) or early CKD stages are limited. Patients with severely reduced renal function are at increased risk of fluid retention and heart failure with ERAs, potentially limiting their use or necessitating dose adjustments and careful monitoring (63). Conversely, early-stage CKD patients may derive greater renoprotective benefits, but clinical trials have not extensively included this population to confirm long-term outcomes. Furthermore, pharmacokinetics of ERAs may be altered in advanced CKD; genetic polymorphisms affecting drug transporters (e.g., OATP1B1) influence plasma drug exposure and response, suggesting that individualized dosing based on renal function and genotype could optimize outcomes (64).

Combination therapies involving ERAs and other agents, such as SGLT2 inhibitors, have shown synergistic effects in reducing albuminuria and mitigating fluid retention, potentially expanding the safe use of ERAs across CKD stages (65). The diuretic effect of SGLT2 inhibitors can counterbalance ERA-associated fluid retention, improving tolerability in patients with compromised renal function. Moreover, ongoing trials are evaluating ERAs in various glomerular diseases and CKD stages, aiming to identify patient subgroups that derive the most benefit with minimal risk (66).

### 5.3 Safety and adverse reaction management of endothelin receptor antagonists

ERAs, particularly selective ETAR blockers, have shown promise in treating diabetic nephropathy and other chronic kidney diseases by mitigating vasoconstriction, inflammation, and fibrosis. However, their clinical application is limited by a significant safety concern: fluid retention, which can precipitate or exacerbate heart failure, especially in patients with advanced CKD or type 2 diabetes mellitus (T2DM). The pathophysiology of fluid retention involves multiple mechanisms. ETAR blockade induces vasodilation of the renal vasculature, altering glomerular hemodynamics and promoting sodium and water retention. Additionally, endothelin-1 (ET-1) modulates tubular sodium reabsorption via ETA and ETB receptors; ETA blockade may disrupt this balance, increasing sodium retention. Some ERAs, such as aprocitentan, have shown fluid retention as the most frequent adverse event in clinical trials for resistant hypertension, underscoring the class effect (67). Understanding these mechanisms is critical for developing risk-mitigation strategies and optimizing the therapeutic use of ERAs in diabetic nephropathy.

Given the propensity of ERAs to induce fluid retention, careful patient selection and fluid management strategies are paramount. Patients with pre-existing heart failure, advanced CKD, or significant volume overload are at increased risk and may require exclusion or close monitoring when considering ERA therapy. Baseline assessment should include evaluation of volume status, cardiac function, and renal parameters. In clinical trials, concomitant use of SGLT2 inhibitors has emerged as a promising strategy to counterbalance fluid retention due to their natriuretic and diuretic effects, helping maintain euvolemia and reduce edema (68). The multidisciplinary approach involving careful patient screening, adjunctive pharmacotherapy, and vigilant monitoring is crucial for the safe implementation of ERAs in diabetic nephropathy management (47).

Long-term administration of ERAs necessitates comprehensive safety monitoring to detect and manage adverse effects, ensuring sustained therapeutic benefit while minimizing harm (69, 70). Although hepatotoxicity is less frequent with newer selective ERAs, periodic liver function tests are warranted. Anemia may result from hemodilution due to fluid retention or direct bone marrow effects and should be monitored via hemoglobin levels (67). Incorporation of precision pharmacovigilance approaches—including pharmacogenetics and real-world data analysis—may enhance individualized risk assessment and management (71). Long-term safety data remain limited, underscoring the need for continued post-marketing surveillance and large-scale clinical trials to establish the risk–benefit profile of ERAs in diverse patient populations. Ultimately, integrating safety monitoring protocols, patient education, and multidisciplinary care will optimize ERA therapy outcomes in diabetic nephropathy (47).

## 6 Combined application of ERA with other drugs and future treatment strategies

### 6.1 Advantages of combination therapy with ERA and RAAS inhibitors

The combination of ERAs with RAAS inhibitors represents a promising therapeutic strategy for DKD by targeting complementary pathogenic pathways to achieve enhanced renal protection. RAAS activation contributes to DKD progression through hemodynamic and pro-fibrotic effects, and while RAAS inhibitors (e.g., ACEIs, ARBs) reduce proteinuria and slow renal decline, many patients experience residual risk. ERAs, particularly selective ETAR antagonists, address additional pathways—notably endothelin-1–mediated vasoconstriction, inflammation, and fibrosis—that are not fully inhibited by RAAS blockade alone. Dual therapy thus provides a more comprehensive mechanism for preserving renal function (47).

Clinical evidence, such as from the SONAR trial, supports the efficacy of this approach. Adding atrasentan to background RAAS inhibitor therapy significantly reduced albuminuria and delayed the composite renal endpoint in DKD patients. These benefits are attributed to synergistic suppression of maladaptive hemodynamic and fibrotic pathways. Importantly, the combination was generally well-tolerated, with fluid retention manageable through patient selection and monitoring (72).

Recent guidelines recognize the value of combining RAAS inhibitors with emerging therapies like ERAs. Furthermore, integrating SGLT2 inhibitors or mineralocorticoid receptor antagonists (MRAs) may offer multi-mechanistic protection, addressing the complex pathophysiology of DKD through hemodynamic, metabolic, and anti-inflammatory effects (73, 74). Based on this, we can conclude that ERA and RAAS inhibitor combination therapy offers synergistic renoprotection in DKD, with clinical trials confirming its potential to improve outcomes beyond standard monotherapy.

### 6.2 Combined potential of ERAs with SGLT2 inhibitors and GLP-1 receptor agonists

Combining ERAs with sodium-glucose cotransporter 2 (SGLT2) inhibitors and glucagon-like peptide-1 receptor agonists (GLP-1 RAs) represents a multi-targeted approach for DKD, leveraging complementary mechanisms. SGLT2 inhibitors reduce glomerular hypertension and hyperfiltration independent of glycemia, while GLP-1 RAs improve glycemic control, promote weight loss, and exert anti-inflammatory effects. ERAs add specific anti-fibrotic and anti-inflammatory benefits via ETAR blockade. Preclinical studies suggest that such combinations may yield additive or synergistic effects, more effectively reducing albuminuria and fibrosis than single agents (74, 75).

**Figure 3:**
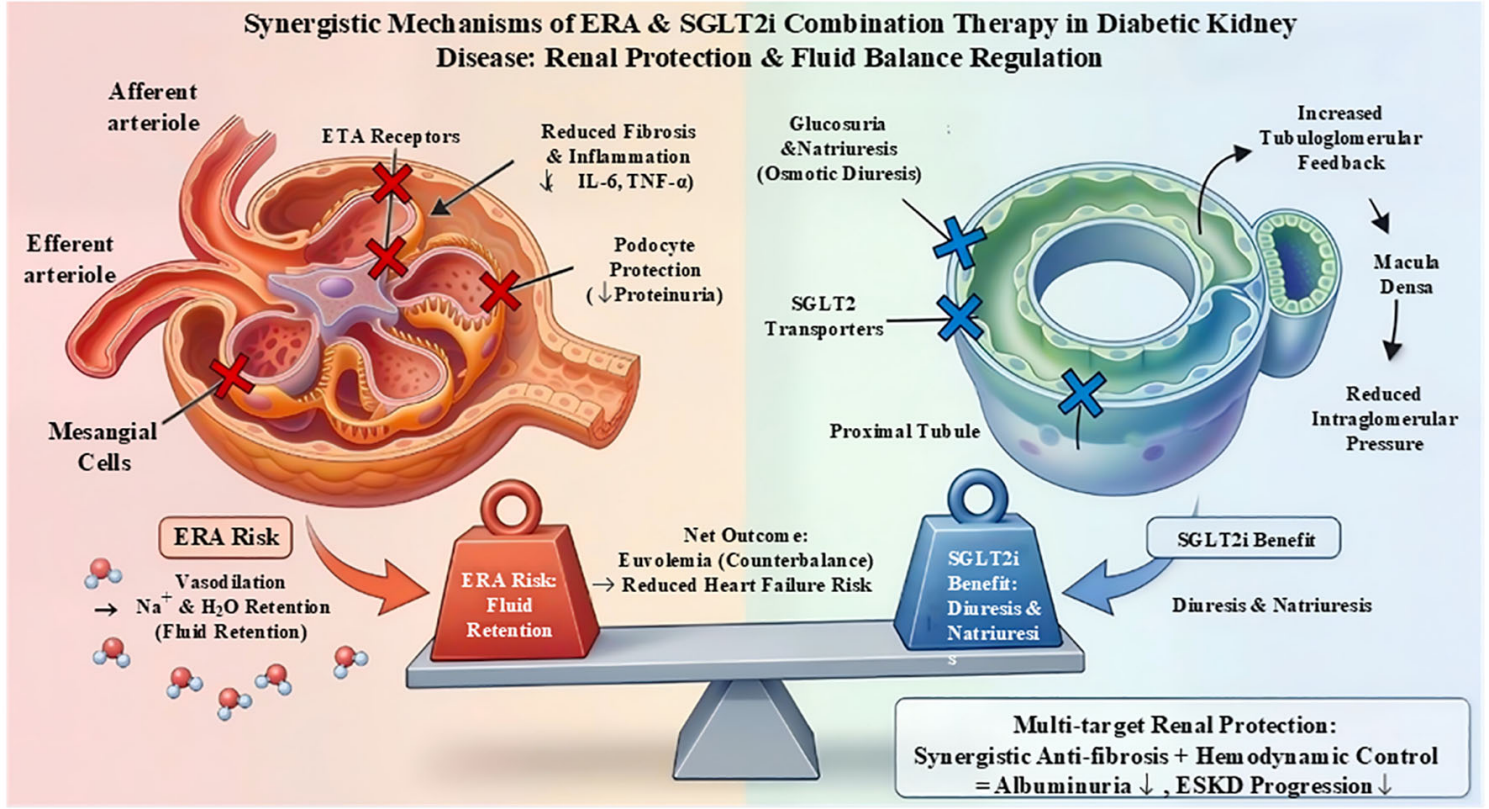
Complementary mechanisms and clinical benefits of combined ETA receptor antagonism and SGLT2 inhibition. ETA receptor antagonists block ETA-mediated signaling in glomerular cells, reducing inflammation, fibrosis, and podocyte injury, but may induce distal tubular sodium retention as a side effect. SGLT2 inhibitors reduce proximal tubular sodium and glucose reabsorption, decrease glomerular hyperfiltration, alleviate tubular hypoxia, and promote osmotic diuresis. Together, combined ERA and SGLT2 inhibitor therapy enhances proteinuria reduction, preserves estimated glomerular filtration rate, and achieves a manageable safety profile through complementary and counter-regulatory mechanisms.

Clinically, SGLT2 inhibitors have demonstrated robust cardiorenal benefits, including slowing CKD progression and reducing heart failure hospitalizations. Their natriuretic and diuretic effects may also counterbalance ERA-associated fluid retention, improving safety (**Figure 3**). GLP-1 RAs contribute additional cardiovascular risk reduction. Early-phase trials, such as those combining zibotentan with dapagliflozin, show enhanced albuminuria reduction with mitigated fluid retention, supporting the feasibility of this approach (62).

This multi-drug strategy aligns with the concept of addressing the cardiovascular-kidney-metabolic (CKM) syndrome as an interconnected entity. By simultaneously targeting metabolic, hemodynamic, inflammatory, and fibrotic pathways, combination therapy may provide comprehensive protection against DKD progression and cardiovascular events (62, 76). Ongoing trials are expected to clarify the efficacy and safety of these regimens, potentially establishing a new standard in DKD management.

### 6.3 Development of new-generation ERA drugs and targeted delivery technologies

Recent advances in ERA development focus on improved receptor selectivity and safety profiles. Newer agents, such as atrasentan and zibotentan, offer high ETAR specificity, preserving ETB-mediated vasodilation and reducing off-target effects. Dual-acting agents like sparsentan (combining ETA and angiotensin receptor blockade) show promise in reducing proteinuria with improved tolerability (77).

In parallel, drug delivery technologies—including nanoparticles, liposomes, and metal-organic frameworks (MOFs)—enable targeted renal delivery, enhancing efficacy while minimizing systemic exposure. For example, nanoMIL-89 has been used to encapsulate bosentan, improving its anti-inflammatory effects while reducing cytotoxicity (78). Biomimetic systems, such as exosome-based carriers, offer further potential for precise kidney targeting.

These innovations may overcome traditional limitations of ERA therapy, such as fluid retention, and improve patient adherence through sustained release. Combining novel ERAs with SGLT2 inhibitors or other nephroprotective agents could further optimize outcomes (25, 28). While challenges in stability and scalability remain, these advances represent a significant step toward personalized, precision medicine in DKD treatment.

## 7 Challenges and Future prospects in the clinical translation of endothelin A receptor antagonists

### 7.1 Major barriers to clinical application

The clinical application of ERAs in diabetic nephropathy (DN) faces significant challenges, primarily related to adverse event management and the difficulty of tailoring therapies to individual patients. Although ERAs such as atrasentan have demonstrated efficacy in reducing albuminuria, lowering blood pressure, and improving lipid profiles, their use is frequently complicated by fluid retention, which can precipitate or exacerbate heart failure—especially in patients with advanced CKD or pre-existing cardiovascular comorbidities (25). Fluid overload remains the most reported adverse effect, often necessitating careful patient selection, dose adjustment, and co-administration of diuretics or SGLT2 inhibitors to mitigate risk (25, 58). Other adverse events, such as anemia and hypoglycemia, further complicate the safety profile of ERAs in this population (43).

The heterogeneity of patient responses— influenced by renal function, cardiovascular status, and insulin resistance—underscores the need for individualized therapeutic strategies. For instance, while atrasentan has shown potential in reducing insulin resistance (potentially contributing to its renoprotective effects), the magnitude of benefit varies among patients (60). Balancing efficacy with safety requires comprehensive monitoring and personalized dosing regimens to optimize outcomes and minimize harm. Despite promising results, these adverse effects remain a substantial barrier to widespread clinical adoption, necessitating ongoing research to refine patient selection criteria and develop protocols for effective side-effect management.

The advancement of ERAs is also hampered by limitations in existing clinical trial designs and a consequent lack of robust evidence regarding long-term efficacy and safety. Most randomized controlled trials (RCTs) conducted to date have been relatively small or of short duration, restricting the generalizability of findings and the ability to detect rare but serious adverse events (58). For example, the pivotal SONAR trial demonstrated significant renal risk reduction with atrasentan but also highlighted challenges related to fluid retention, indicating that larger-scale studies are needed to optimize dosing and patient selection (43). Furthermore, many trials have relied on surrogate endpoints (e.g., albuminuria reduction) rather than hard clinical outcomes such as progression to end-stage renal disease or cardiovascular mortality, limiting the clinical interpretability of the results (61).

The heterogeneity of DN phenotypes and the influence of concomitant therapies—such as RAAS inhibitors and SGLT2 inhibitors— further complicate the isolation of ERA-specific effects. This necessitates more sophisticated trial designs incorporating stratified or precision medicine approaches (79). Additionally, the exclusion of patients with advanced heart failure or severe CKD from many trials restricts understanding of ERA safety in these high-risk groups. Ongoing and future Phase 3 trials, including those investigating dual receptor blockers like sparsentan, aim to address these gaps by enrolling larger, more diverse populations and employing longer follow-up periods to better characterize efficacy and safety profiles (55). Until such data are available, the clinical translation of ERAs remains cautious, underscoring the importance of continued rigorous research to overcome current evidence limitations.

### 7.2 Precision medicine and biomarker-guided optimization of ERA therapy

The optimization of endothelin receptor antagonist (ERA) therapy through precision medicine approaches represents a promising frontier in the management of DKD. Leveraging multi-omics technologies and biomarker integration allows treatments to be tailored to individual patient profiles. The heterogeneity of DKD pathogenesis— characterized by diverse molecular and cellular mechanisms such as hyperglycemia-induced oxidative stress, endothelial dysfunction, podocyte injury, inflammation, and fibrosis—necessitates patient stratification beyond traditional clinical parameters (80, 81).

Multi-omics platforms—including transcriptomics, proteomics, metabolomics, and lipidomic—enable comprehensive molecular profiling of kidney tissue and biofluids, facilitating the identification of distinct DKD subtypes that may respond differently to ERA therapy. For example, transcriptomic analyses can reveal gene expression signatures linked to endothelin pathway activation or endothelial-podocyte axis disruption, helping pinpoint patients most likely to benefit from selective ETAR blockade (80, 82). Proteomic studies complement this by quantifying circulating or urinary proteins reflective of glomerular injury, fibrosis, or inflammation, serving as surrogate markers for treatment responsiveness and disease progression (81).

Biomarkers are critical not only for patient selection but also for monitoring therapeutic efficacy and safety. While established biomarkers such as proteinuria and eGFR remain essential, emerging biomarkers from multi-omics data offer improved sensitivity and specificity. For instance, biomarkers indicative of endothelial dysfunction (e.g., circulating ET-1 levels) or podocyte injury may predict the degree of proteinuria reduction achievable with ERA therapy (80, 83). Additionally, biomarkers could aid in the early detection of adverse effects such as fluid retention and heart failure risk, which have historically limited ERA use. Integrating biomarker monitoring into clinical protocols allows dynamic treatment adjustments, optimizing the balance between efficacy and safety (84).

Initiatives such as the Kidney Precision Medicine Project exemplify the application of artificial intelligence and machine learning to integrate multi-omics data with clinical phenotypes, refining DKD classification and guiding individualized treatment strategies, including ERA use (82). These approaches hold potential of identifying novel mechanism-based DKD subtypes, enabling targeted interventions that address underlying molecular drivers rather than merely managing symptoms. Moreover, combining ERAs with other agents (e.g., SGLT2 inhibitors, MRAs), guided by biomarker profiles, may yield synergistic benefits by concurrently modulating distinct pathogenic pathways (85).

### 7.3 Future research directions and clinical trial design recommendations

The current landscape of endothelin A receptor antagonist (ERA) research underscores the need for well-designed multicenter randomized controlled trials (RCTs) with large sample sizes and extended follow-up periods. Although trials such as SONAR have demonstrated that selective ERAs (e.g., atrasentan) can reduce albuminuria and slow CKD progression in type 2 diabetes, the generalizability of these findings remains limited due to sample size constraints, patient heterogeneity, and relatively short durations of follow-up (43, 58). Larger multicenter studies are essential to confirm long-term efficacy and safety across diverse populations, including patients with varying degrees of renal impairment and comorbidities. Extended follow-up is particularly important for evaluating sustained renal protection, cardiovascular outcomes, and adverse events such as fluid retention and heart failure, which are commonly associated with ERA therapy (25, 47).

Future trials should incorporate stratification by sex and other demographic factors, especially since *post-hoc* analyses of SONAR revealed sex-based differences in treatment response and heart failure risk (57). The inclusion of robust biomarkers and surrogate endpoints—such as changes in eGFR, albuminuria, and inflammatory markers—will enhance mechanistic understanding and facilitate early detection of treatment effects and adverse outcomes (86).

Combination therapies targeting complementary pathogenic pathways represent a promising strategy given the multifactorial nature of DN and CKD. ERAs have shown additive benefits when combined with RAAS inhibitors, SGLT2 inhibitors, and mineralocorticoid receptor antagonists (MRAs) (62). These combinations may enhance antiproteinuric and renoprotective effects while mitigating adverse events such as fluid retention through mechanisms like the diuretic action of SGLT2 inhibitors (62). However, the safety profiles of combination regimens require rigorous evaluation, particularly concerning risks of volume overload, hyperkalemia, and cardiovascular events. Although clinical trials to date have provided encouraging preliminary data, further studies are needed to systematically assess adverse events, optimal dosing, and patient phenotypes most likely to benefit from combination therapy (25, 47).

Future research should prioritize the integration of novel ERAs with improved selectivity and safety profiles into personalized treatment paradigms. Recent advances include highly selective ETA antagonists such as zibotentan and dual-acting agents like sparsentan, which exhibit promising efficacy with reduced risk of fluid retention (25, 55). The combination of low-dose ERAs with SGLT2 inhibitors has also shown synergistic antiproteinuric effects with improved tolerability (55). To harness these advances, future clinical trials should incorporate biomarker-driven patient stratification, leveraging molecular and histopathological phenotyping to identify responders and tailor therapy accordingly (79, 87). Emerging insights into the role of endothelin-1 in podocyte injury, endothelial dysfunction, and inflammation provide mechanistic bases for targeted interventions and companion diagnostics (80). Furthermore, integrating pharmacogenomic data and clinical decision-support systems can facilitate individualized dosing and monitoring, minimize adverse effects, and maximize efficacy (88). The ongoing expansion of multi-omics and imaging technologies will further refine patient selection and therapeutic monitoring. Ultimately, the future of ERA therapy lies in a precision medicine framework that combines novel agents, optimized combination regimens, and individualized treatment algorithms to improve renal and cardiovascular outcomes in diabetic nephropathy.

## 8 Conclusion

The endothelin-1–ETA axis represents a critical mechanistic pathway in the progression of diabetic kidney disease. ETA receptor antagonists have demonstrated consistent antiproteinuric and renoprotective effects in both preclinical and clinical studies. However, safety concerns, particularly fluid retention, remain a key limitation.

Future therapeutic strategies are likely to involve biomarker-guided patient selection and combination therapy with agents such as SGLT2 inhibitors to enhance efficacy and minimize adverse effects. Further large-scale, long-term trials are required to define the optimal clinical role of ERAs in DKD management.
